# Knowledge and attitudes about vitamin A consumption and its relationship with night blindness in university students

**DOI:** 10.3389/fmed.2024.1309837

**Published:** 2024-04-26

**Authors:** Suparmi Suparmi, Harka Prasetya, Atik Rahmawati, Millam Shinta Lailaulaan

**Affiliations:** ^1^Department of Biology, Faculty of Medicine, Universitas Islam Sultan Agung, Semarang, Indonesia; ^2^Department of Ophthalmology, Faculty of Medicine, Universitas Islam Sultan Agung, Semarang, Indonesia; ^3^Sultan Agung Eye Center, Rumah Sakit Islam Sultan Agung, Semarang, Indonesia; ^4^Faculty of Medicine, Universitas Islam Sultan Agung, Semarang, Indonesia

**Keywords:** vitamin A deficiency, night blindness, education, knowledge, attitudes

## Abstract

**Introduction:**

Night blindness is the first sign of vitamin A deficiency (VAD), which can lead to blindness if left untreated. University students may be at risk of VAD-related night blindness due to unhealthy eating attitudes and inadequate vitamin A intake. This study aimed to determine the relationship between knowledge and attitudes toward vitamin A consumption affecting night blindness in university students.

**Methods:**

This cross-sectional study involved 409 third-year university students of Universitas Islam Sultan Agung, Semarang, Indonesia. Participants completed questionnaires about socio-demographics, their knowledge of vitamin A, and attitudes toward vitamin A consumption. Night blindness symptoms among university students were assessed using the Low Luminance Questionnaire (LLQ), followed by a bivariate analysis of the Chi-Square test. Multivariate binary logistic regressions were used to determine whether the independent variables were associated with night blindness. A *p*-value less than 0.05 indicated significance.

**Results:**

The prevalence of high-symptom night blindness was higher among males (26.4%) than females (5.7%). Out of 409 university students, 48 from the non-medicine cluster of the study program had a night blindness symptom. The prevalence was lower in students who studied in the medicine cluster program. The level of knowledge on vitamin A had a significant relationship with symptoms of night blindness [prevalence ratio (PR) = 2.239 (95% CI = 1.110–4.516)]. The attitudes toward vitamin A consumption were significantly associated with night blindness (PR = 2.560, 95% CI = 1.215–5.392).

**Discussion:**

The results of this study show that the risk of night blindness in university students can be prevented by increasing their knowledge and attitudes toward consuming vitamin A-rich food. The university can provide health promotion and vitamin A supplementation to avoid night blindness among academia.

## Introduction

Vitamin A is an essential nutrient that is crucial in maintaining eye health. Vitamin A’s active all-trans retinol metabolite endures a cycle that generates rhodopsin for light perception in the eye (1). Rhodopsin is involved in the dark adaptation process, allowing humans to see in the faint light. People with night blindness can see well during the day due to their cone cells, but they have difficulty seeing at night due to the ineffectiveness of their rod cells. Vitamin A deficiency (VAD) causes night blindness, resulting in the challenges of performing tasks in faint light or at night (1, 2).

Night blindness is the first symptom of VAD, which can lead to blindness if left untreated (3–5). Consuming foods deficient in vitamin A is common in developing countries (6, 7) due to poor eating habits. University students tend to consume high-calorie food selections, fast food snacks, fried food, and a low intake of daily fruits and vegetables (8). The low intake of vitamin A-rich foods among university students can increase the risk of ocular health disorders (9–13), especially night blindness.

Previous studies focus on vitamin A, and conclusive findings indicate that many university students suffer from vitamin A deficiency. The average daily intake of vitamin A among university students was about 2,500–5,000 IU. The treatments of 3 capsules containing 100,000 IU improved in dark adaptation, as indicated by the decrease of the final rod threshold of more than 0.15 log μμl (14). A recent study by Wan et al. reported no vitamin A deficiency in college students; however, all the students are vitamin E deficient (15). Knowledge, attitudes, and practices of medical students regarding vitamin consumption have been studied in China (16, 17), but we did not find vitamin A deficiency in medical students in Indonesia and its effects on night blindness symptoms.

Preventing VAD in university students, especially medical students, is vital in reducing errors in identifying color slides and specimens and investigating specific physical signs (18). The serial monitoring of electroretinogram (ERG) testing showed that vitamin A supplementation could restore VAD-associated night blindness in patients with nyctalopia complaints (19). Therefore, precautionary measures can be taken to improve knowledge and attitudes toward consuming vitamin A (20). This study aims to determine the relationship between the level of knowledge and attitudes toward vitamin A-rich foods consumption and night blindness symptoms in university students.

## Materials and methods

### Study design, population, and sample

This research is an analytical observational study with a cross-sectional design. Nine faculties at the Universitas Islam Sultan Agung, Semarang, Indonesia, involved in this study, categorized into 2 clusters. The study program of the Medicine cluster consisted of students enrolled in the Faculty of Medicine, Faculty of Dentistry, and Faculty of Nursing. In contrast, the non-medicine cluster included the Faculty of Law, Faculty of Islamic Studies, Faculty of Languages and Communication Sciences, Faculty of Engineering, and Faculty of Industry Engineering. The inclusion criteria were university students in 3rd-year bachelor study, while the exclusion criteria were missing written consent and night blindness before university. The sample size was 368, calculated using the following equation (21); where n is the minimum sample size required in the study, Z is the area under the curve corresponding to the desired confidence interval used in this study, i.e., 95% CI (1.96), P is the prevalence of vitamin A deficiency (VAD) in Indonesia (60%) (22), and d is the precision [difference between sample mean and population mean (+/− 5%)]. Assuming adding 10% of the sample size, 409 university students with bachelor’s degrees were involved in this study.


$$n=\frac{N.Z_{1-{\frac{a}{2}}^{2}}.p.\left(1-p\right)}{d^{2}}$$

### Data collection tool and measurement

The data collection was performed on 1–31 July 2022. All research instruments, including the consent forms and questionnaires, were available in Indonesian. The questionnaires were arranged in a Google form and shared through a WhatsApp group of students. The students filled out the questionnaire, and we provided online guidance when necessary.

The self-administered structured questionnaire consisted of sociodemographic characteristics (gender, age, and faculty), vitamin A knowledge, and attitudes toward vitamin A consumption (23). A knowledge-level questionnaire on vitamin A consumption consists of 20 questions about vitamin A deficiency’s sources, benefits, and consequences (Table 1) with the response of True or False. The level of knowledge is categorized as low-middle if the number of correct answers is 0–13 and high if the right answers are 14–20. Vitamin A consumption attitudes were asked through a questionnaire with 20 questions (Yes or No responses) related to the type, frequency, and habits of consuming foods that contain vitamin A (Table 2). Attitudes are categorized as low-middle (0–13 correct answers) and high (14–20 correct answers). Before conducting the research, the self-designed questionnaire was administered to 50 randomly selected university students for comprehension testing to ensure validity and reliability. The validity test showed that the corrected item-total correlation was >0.279, while the reliability test showed that Cronbach’s Alpha values of all items were > 0,279. Therefore, no significant changes were made due to the preliminary examination.

**Table 1:** The questions on knowledge regarding vitamin A administered to university students (*n* = 409).

No	Question
1	Vegetables that are green and yellow are rich sources of vitamin A
2	Vitamin A serves various physiological roles in the body, including its involvement in the visual system
3	Vitamin A is essential for the maintenance of optimal eye health.
4	Vitamin A deficiency can lead to a condition known as night blindness.
5	Vitamin A deficiency can lead to blindness.
6	Vitamin A is essential for optimal visual acuity in well-lit conditions.
7	Prolonged intake of high amounts of vitamin A might lead to ocular impairment.
8	Vitamin A is essential for the formation and growth of bones.
9	Vitamin A is essential for bone marrow formation.
10	Vitamin A plays a role in the production of erythrocytes.
11	Vitamin A enhances physical stamina.
12	Vitamin A stimulates the desire to eat.
13	Utilization of Vitamin A, together with vitamins C and E, can prevent cancer.
14	The consumption of vitamin A, in addition to vitamins C and E, can effectively prevent heart disease.
15	Consuming vitamin A can promote reproductive growth.
16	The consumption of vitamin A can enhance the body’s immune system.
17	Consumption of Vitamin A is effective in preventing blindness.
18	Vitamin A can impact cognitive function
19	Dietary vitamin A promotes overall health and enhances physical strength.
20	Consuming vitamin A can reduce the occurrence of heightened diarrhea.

**Table 2:** The questions on attitudes regarding vitamin A administered to university students (*n* = 409).

No	Question
1	I consume a diverse range of verdant vegetables.
2	I consume a diverse range of yellow vegetables.
3	I consume food that has been prepared using vegetable oil.
4	I refrained from consuming fruit due to its potential to contribute to weight gain.
5	Given the choice, I prefer consuming cake over fruit or liquids.
6	If veggies are present in the household, I autonomously acquire and consume them without instruction.
7	Consuming fruit in the morning is beneficial as it supplies ample energy to sustain until midday.
8	I consume green vegetables on a daily basis.
9	Daily basis, I consume yellow veggies and fruits.
10	I consume fruit daily.
11	I prefer consuming unhealthy food over consuming fruits and vegetables.
12	Economical fruits such as bananas, pineapples, and peppers have limited vitamins.
13	From my perspective, fruit is a nutritious and beneficial dietary option.
14	I enjoy consuming uncooked green vegetables.
15	I enjoy consuming uncooked yellow vegetables.
16	I like consuming green vegetables than fried chicken.
17	I like consuming yellow vegetables than fried chicken.
18	I must consume a diverse range of meals daily.
19	I abstain from consuming food that includes veggies.
20	I consume fruits and vegetables due to their plentiful availability.

Symptoms of night blindness were assessed by the Low Luminance Questionnaire (LLQ). This LLQ is a valid and reliable test for patient-centered assessment of visual function in a low luminance or mesopic setting (24, 25). The questionnaire was initially accessed in English and modified into Bahasa Indonesia (the national language). The LLQ is a 32-item questionnaire with six subscales related to low luminance settings: driving, mobility, extreme lighting, general dim lighting, and peripheral vision (Table 3). Each question can be answered with Yes or No. Symptoms are categorized as low-middle if the number of “Yes” answers is 0–21 and high for 22–32.

**Table 3:** The questions on symptoms of night blindness using the Low Luminance Questionnaire (LLQ) administered to university students (*n* = 409).

No	Respondent characteristic
1	I have difficulty seeing in bright sunlight.
2	I have difficulty seeing in fluorescent lighting, like that found in stores and offices.
3	I have difficulty seeing people’s faces in a hallway when direct sunlight is behind them.
4	I have difficulty reading menus in dimly lit restaurants.
5	I have difficulty reading the newspaper without good lighting.
6	I get upset because I have difficulty seeing while driving in the rain at night.
7	I have difficulty in comprehending text that is printed on dark-colored paper.
8	I have difficulty seeing dark colored cars while driving at night.
9	Because of my vision, I am bothered that I have difficulty moving around in a darkened theater.
10	Because of my vision, I have difficulty going out to nighttime social events such as sporting events, the theater, friend’s homes, church, or restaurants.
11	I depend on others to help me because of my vision at night or under poor lighting.
12	I worry or concerned that I might fall at night because of my vision.
13	I have difficulty seeing colors at night.
14	I have difficulty seeing furniture in dimly lit rooms with dark floors.
15	I have difficulty seeing at night.
16	I have difficulty seeing in poor lighting conditions such as at dusk or dawn or in a poorly lit room.
17	I have difficulty perceiving the depth of the basin during nighttime.
18	I have difficulty seeing in candlelight,
19	I have difficulty seeing when you visit other people’s homes because there is not enough light.
20	I have difficulty seeing under kitchen counters or in cabinets or closets because there is not enough light.
21	I have difficulty with my peripheral vision under poor lighting conditions.
22	I have difficulty with my peripheral vision at night.
23	I have difficulty with my peripheral vision in bright sunlight
24	I have difficulty reading street signs when driving at night.
25	While driving at night, I have difficulty headlighting from oncoming cars.
26	I have limited driving in the rain at night because of difficulty seeing.
27	I limit your driving at night due to my vision.
28	I have difficulty seeing while driving at dawn or dusk because of glare.
29	I worry or I am concerned that I may make a mistake at a social event because I cannot see well enough under poor lighting conditions (for example, getting food on a fork, recognizing people, or reading the menu in a dimly lit restaurant).
30	I feel bad or depressed about my ability to see at night or under poor lighting conditions.
31	I feel bad or depressed because my vision at night or under poor lighting keeps me from doing all that me would like to do.
32	I feel bad or depressed that I am not able to help others as much as I want because of my vision at night or under poor lighting.

### Data analysis

Data were analyzed using IBM SPSS Statistics version 25.0 for Windows. A bivariate analysis of the chi-square test was carried out to explore the relationship between knowledge and attitudes toward vitamin A consumption with the appearance of symptoms of night blindness. Multivariate analysis of logistic regression was used to determine the most influential variables on the signs of night blindness.

## Results

Table 4 shows the sociodemographic data of the participants. A total of 409 university students participated in this study, of which 47.2% were female and 52.8% were female. A total of 56% respondents studied at the Medicine Cluster Study Programme (Medicine, Dentistry, and Nursing), while 44% were enrolled in the Non-Medicine Cluster (Islamic Studies, Law, Language and Communication Sciences, Economics, Electrical Engineering, and Industrial Engineering).

**Table 4:** Sociodemographic characteristics of respondents (*n* = 409).

Respondent characteristic	n (%)
Sex	
Female	193 (47.2)
Male	216 (52.8)
Age	
18–21 years old	200 (48.8)
22–25 years old	209 (51.2)
The cluster of study program	
Medicine	229 (56.0)
Non-medicine	180 (44.0)

The prevalence of high symptoms of night blindness was higher among males (26.4%) than females (5.7%). The prevalence of night blindness did not differ significantly between the two age groups (*p* > 0.05). Out of 180 university students from the non-medicine cluster of the study program, 48 (26.7%) students had a night blindness symptom. The prevalence was lower in students who studied in the medicine cluster of the study program (8.7% out of 229 students) (Table 5). The level of knowledge on vitamin A had a significant relationship to the symptoms of night blindness with a prevalence ratio (PR) of 2.239 (95% CI = 1.110–4.516). However, it can be seen that 19% out of 295 students with a high level of knowledge about vitamin A showed a symptom of night blindness. The attitudes toward vitamin A consumption were significantly associated with night blindness (PR = 2.560, 95% CI = 1.215–5.392). However, 21.2% of the 273 students with high attitudes toward vitamin A consumption showed high symptoms of night blindness.

**Table 5:** The relationship between sex, age, a cluster of study program level of knowledge about vitamin A and attitudes toward vitamin A consumption and night blindness symptom.

Variables	Night blindness symptom [n, (%)]		p*	PR (CI)
	Low-middle	High		
Sex			<0.001	3.764 (1.725–8.213)
Female	182 (94.3)	11 (5.7)		
Male	159 (73.6)	57 (26.4)		
Age			0.097	0.553 (0.235–1.305)
18–21 years old	173 (86.5)	27 (13.5)		
22–25 years old	165 (80.4)	41 (19.6)		
A cluster of the study program			<0.001	2.003 (1.030–3.896)
Medicine cluster	209 (91.3)	20 (8.7)		
Non-medicine cluster	132 (73.3)	48 (26.7)		
Level of knowledge about vitamin A			0.039	2.239 (1.110–4.516)
Low-middle	102 (89.5)	12 (10.5)		
High	239 (81.0)	56 (19.0)		
Attitudes toward vitamin A consumption			<0.001	2.560 (1.215–5.392)
Low-middle	126 (92.6)	10 (7.4)		
High	215 (78.8)	58 (21.2)		

*Bivariate analysis of the chi-square test.

## Discussion

This cross-sectional study aimed to determine the association between knowledge and attitudes on vitamin A consumption and night blindness symptoms among university students. Similar studies have been carried out in Indonesia to determine the relationship between the knowledge of mothers and attitudes toward vitamin A supplementation to their children (22, 26, 27). However, in this study, we focus on the prevalence of night blindness-related vitamin A deficiency (VAD) due to unhealthy eating attitudes with inadequate vitamin A intake in university students. College students may experience hidden malnutrition for certain essential nutrients, including vitamin A and vitamin E (28).

This study reported that the prevalence of night blindness was five times higher in male than female students. Similar results were also seen in a cross-sectional study by Faruqui et al., who reported that the prevalence of color vision deficiency (CVD) in male medical students was higher than in female medical students (29). The risk of blindness tends to be higher in men due to a mutation in the nyctalopia gene (NYX) on Xp11.4 and X chromosome-related deficiencies in men (30). Night blindness in male students may also led by screen time. The prevalence of night blindness did not differ significantly between the two age groups because the participants were enrolled in the same year of their bachelor’s study. However, in children, age was mainly related to the prevalence of night blindness (31). Another study by Shrestha et al. reported that low awareness of common ocular conditions, including night blindness, is associated with factors such as female gender, old age, lower levels of education, and rural habitation. Thus, health literacy promotion can be helpful for eye care services (32).

The participants with a low-middle level of knowledge on vitamin A had two times higher risk of night blindness symptoms. It can also be seen from the result of the association between the cluster of the study program and night blindness. The university students from the non-medicine cluster of the study program had a higher prevalence of night blindness symptoms (26.7% out of 180 students) than those who studied in the medicine cluster of the study program (8.7% out of 229 students). It can be caused by the different knowledge about vitamin A during their study. Changing practices requires continuous long-term education (33). Rachman et al. reported that fruit and vegetable consumption behavior among senior high schools in Bali, Indonesia, was related to attitude, nutritional knowledge, food availability, media exposure, and parent’s income (34). People with a greater understanding of vitamin A are typically more aware of vitamin A’s benefits for eye health; thus, education in society is needed to improve knowledge, attitudes, and practices toward vitamin A consumption (23).

Nineteen percent out of 295 students with a high level of knowledge about vitamin A and 8.7% out of 209 students from the medicine cluster of study program showed a symptom of night blindness. We speculate that they could have suffered from night blindness due to stress, heavy workload, and lack of time; university students tend to make poor food choices. Thus, it is challenging for them to adhere to vitamin A-rich food. A student enrolled in the medicine cluster of the study program has a stressful environment due to the extensive curricula, numerous academic requirements, and frequent, complex, and various types of examinations (35). It can also be caused by their long screen time. Dixit et al. reported that screen time was associated with adolescents’ increasing prevalence of poor, uncorrected visual acuity (36).

The attitudes toward vitamin A consumption were significantly associated with night blindness (odds ratio = 2.560, 95% CI = 1.215–5.392). However, 21.2% of the 273 students with high attitudes toward vitamin A consumption showed high symptoms of night blindness. This study is consistent with a previous study stating that attitudes did not always translate into practice; for example, although 60.5% agreed on the adverse effect of interruption during mealtime on food intake, they do not interrupt a patient during mealtime (37). Adequate vitamin A supplementation is reported to be an effective treatment of night blindness because vitamin A deficiency tends to be reversible (5). The Food and Nutrition Board, Institute of Medicine—National Academy of Sciences advises that the Recommended Daily Allowance (RDA) for >18 years of age people is 700 mg/d for females and 900 mg/d as retinol activity equivalents (RAE’s), while the Tolerable Upper Intake Level (ULs) for males is 3,000 mg/d (38).

This study was subject to several limitations. First, this study was a cross-sectional design; thus, it is difficult to determine whether the low level of knowledge and attitudes of vitamin A preceded the night blindness. Second, data collection by questionnaire may have led to recall bias and misreporting of vitamin A-rich food consumption. Regarding the attitude questionnaires toward vitamin A consumption, no quantitative data on daily vitamin A-rich food portions or vitamin A supplementation was available to identify the association between the practices and night blindness of university students. Furthermore, unfortunately, we do not physically observe the night blindness symptoms or measure the serum retinol level of our respondents. Although this study represents a private university in Semarang, Indonesia, and thus is not representative of all university students, our study was the first to show vitamin A consumption among university students and its association with night blindness in Indonesia.

## Conclusion

The results of this study show that the risk of night blindness in university students can be prevented by increasing their knowledge and attitudes toward consuming vitamin A-rich food. Continuous education on the role of a healthy diet is needed to educate undergraduate students, especially those studying in the non-medicine cluster of the study program. To improve this practice, the university can provide health promotion and vitamin A supplementation to prevent night blindness in academia.

## Data availability statement

The original contributions presented in the study are included in the article/supplementary material, further inquiries can be directed to the corresponding author.

## Ethics statement

The studies involving humans were approved by the Institutional Review Board (or Ethics Committee) of the Faculty of Medicine at Universitas Islam Sultan Agung. The studies were conducted in accordance with the local legislation and institutional requirements. The participants provided their written informed consent to participate in this study.

## Author contributions

SS: Conceptualization, Data curation, Formal analysis, Funding acquisition, Investigation, Methodology, Project administration, Resources, Software, Validation, Visualization, Writing – original draft, Writing – review & editing. HP: Funding acquisition, Methodology, Supervision, Writing – review & editing. AR: Methodology, Supervision, Writing – review & editing. ML: Data curation, Formal analysis, Investigation, Writing – review & editing.
